# Non-invasive large-scale imaging of concurrent neuronal, astrocytic, and hemodynamic activity with hybrid multiplexed fluorescence and magnetic resonance imaging (HyFMRI)

**DOI:** 10.1038/s41377-025-02003-9

**Published:** 2025-09-25

**Authors:** Zhenyue Chen, Yi Chen, Irmak Gezginer, Qingxiang Ding, Hikari A. I. Yoshihara, Xosé Luís Deán-Ben, Ruiqing Ni, Daniel Razansky

**Affiliations:** 1 Institute of Precision Optical Engineering, School of Physics Science and Engineering, Tongji University, Shanghai, China; 2 Institute for Biomedical Engineering and Institute of Pharmacology and Toxicology, Faculty of Medicine, University of Zurich, Zurich, Switzerland; 3 Institute for Biomedical Engineering, Department of Information Technology and Electrical Engineering, ETH Zurich, Zurich, Switzerland; 4 Zurich Neuroscience Center (ZNZ), Zurich, Switzerland; 5 Department of Nuclear Medicine, Inselspital, Bern, Switzerland

**Keywords:** Imaging and sensing, Photonic devices

## Abstract

A critical gap currently exists in systematic understanding and experimental validation of the role of astrocytes in neurovascular coupling and their functional links with other brain cells. Despite a broad selection of functional neuroimaging tools for multi-scale brain interrogations, no methodology currently exists that can discern responses from neural and glial cells while simultaneously mapping the associated hemodynamic activity on a large scale. We present a hybrid multiplexed fluorescence and magnetic resonance imaging (HyFMRI) platform for measuring neuronal and astrocytic activity registered to concurrently recorded brain-wide hemodynamic responses. It features a fiberscope-based imaging system for multichannel fluorescence and optical intrinsic signal recordings and a custom surface radiofrequency coil, which are incorporated into the bore of a preclinical magnetic resonance imaging (MRI) scanner. We used HyFMRI to study peripheral-stimulus-evoked brain responses in mice differentially labeled with RCaMP and GCaMP genetically-encoded calcium indicators. Stimulation-evoked neuronal responses displayed the fastest kinetics and highest activation amplitude followed by astrocytic signals and the hemodynamic responses simultaneously recorded with functional MRI. In addition, the activation traces from neurons and astrocytes exhibited high linear correlation, thus providing direct evidence of astrocytic mediation in neurovascular coupling. This newly developed capacity to capture cell-type-specific calcium signaling alongside whole-brain hemodynamics enables the simultaneous investigation of neuro-glial-vascular interactions in health and disease. HyFMRI thus expands the current neuroimaging toolbox for a wide range of studies into synaptic plasticity, neural circuitry, brain function and disorders.

## Abstract (web summary)

This work presents a hybrid fluorescence and magnetic resonance imaging (HyFMRI) platform for concurrent measurements of neuronal, astrocytic, and brain-wide hemodynamic responses in mice.

## Graphical abstract

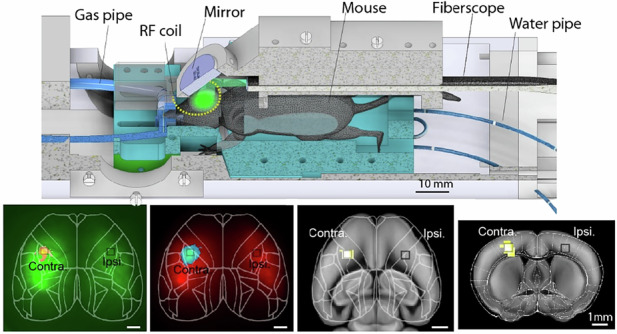

## Introduction

Brain is the most complex and dynamic organ in the body, orchestrating a vast array of functions that underpin cognition, sensation, movement, and emotion. Each signaling event in the nervous system is a carefully coordinated process involving diverse cell types, including neurons, astrocytes, pericytes, smooth muscle cells, and many others, which interact through intricate signaling pathways^1^. To better understand brain function or pathophysiology, it is essential to record multiparametric signals from different cell types given the highly complex and dynamic features of neural processes. This task is however challenged by multiple factors, such as lack of suitable contrast mechanisms, performance limitations of existing neuroimaging techniques, and incompatibilities between different modalities^2^.

Genetically encoded calcium indicators (GECIs) have profoundly transformed neuroscience by enabling the visualization and quantification of calcium dynamics within neurons and other cell types^3,4^. Since calcium ions (Ca^2+^) play a crucial role in the generation and propagation of action potentials and in synaptic transmission, real-time monitoring of their influxes can provide direct insights into neuronal firing patterns and synaptic activity^5^. Ca^2+^ ions also play a central role in the functional regulation of astrocytes, which are critical for maintaining brain homeostasis and provide structural and metabolic support to neurons as well as regulation of synaptic activity^6^. The development of GECIs with distinctive absorption and emission wavelengths has enabled comprehensive studies involving diverse cell types and signaling pathways. Among them, GCaMP and RCaMP exhibit improved sensitivity and dynamic range and have been extensively exploited in studies of neural function, synaptic plasticity, and brain disorders^3,7^.

In parallel, advances in multiplexing and multimodal imaging technologies have greatly enhanced the ability to capture and analyze complex neural activity^8–12^. By detecting blood oxygen level dependent (BOLD) signals, functional magnetic resonance imaging (fMRI) has enabled studying function and structure at the whole-brain level, both in animals and humans. Combining calcium imaging via fiber photometry with fMRI has further allowed for the direct measurement of local neuronal activity and the associated hemodynamic responses, providing a powerful tool for studying the intricate relationships between neurons and glial cells as well as broader aspects of brain function^13,14^. However, fiber photometry lacks the spatial resolution component as it merely collects averaged signal from cells enclosed within a tiny field of view (FOV) surrounding the fiber tip. Conversely, widefield fluorescence (FL) imaging can map neural activity across the entire cortex^15–18^. Yet, its integration with fMRI is not straightforward due to electromagnetic interference inside the MRI scanner. To this end, optical recordings and fMRI from the same subject have mainly been performed sequentially, making it challenging to link measurements across spatial and temporal scales and modalities^19,20^. Concurrent whole-cortex GCaMP imaging and fMRI has been reported^21^ but the system employed a sophisticated telecentric lens system with a camera housed in an adjacent room, which resulted in suboptimal image quality for both modalities while only GCaMP signals from neurons could be recorded.

To address the need for concurrent calcium imaging of multiple cell types in conjunction with whole-brain fMRI, we present a new approach for hybridizing multiplexed optical and MRI recordings (HyFMRI). The platform incorporates a fiberscope-based optical (multichannel FL and optical intrinsic signal) imaging system and a custom surface radiofrequency (RF) coil into the bore of a preclinical MRI scanner. Custom 3D-printed components were designed to bridge the two modalities and provide animal support during the experiment. We then demonstrate concurrent measurements of neuronal, astrocytic, and hemodynamic activity in lightly anesthetized mice and further assess the kinetics and spatiotemporal correlations of electrical stimulation-evoked activity obtained from the concurrent recordings.

## Results

### Hybrid fluorescence and magnetic resonance imaging (HyFMRI) platform

**Fig. 1:**
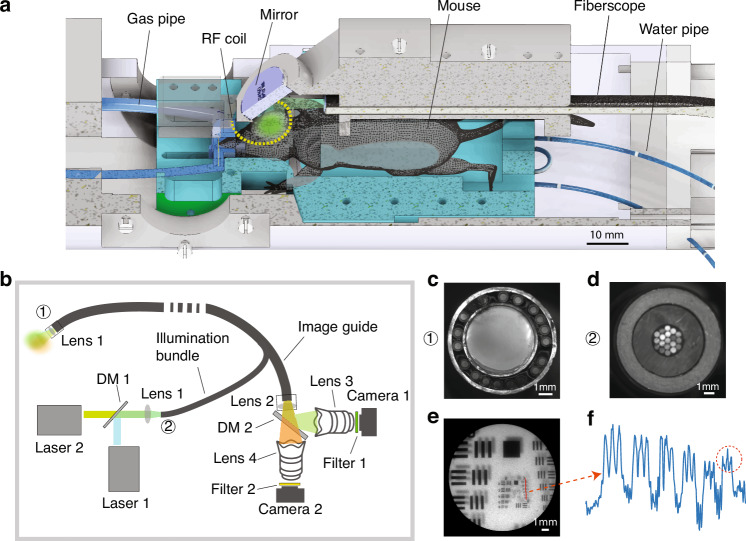
Hybrid multiplexed fluorescence and magnetic resonance imaging (HyFMRI) platform. **a** Illustration of the HyFMRI design comprised of a fiberscope-based fluorescence imaging system, a radio frequency (RF) surface coil and a 3D-printed mouse holder. **b** Schematic drawing of the fiberscope-based fluorescence imaging system. **c** Photograph of the fiberscope tip comprised of an objective in the center surrounded by 19 illumination fibers. **d** Photograph of the input end of the illumination bundle. **e** The effective 15 mm diameter FOV and spatial resolution characterization of the optical imaging system using the 1951 USAF target under white light illumination. **f** Group 3, element 6 in the 1951 USAF target can be resolved, corresponding to a spatial resolution of ~70 μm

The HyFMRI platform was devised by integrating a customized multichannel fiber-optic imaging system with a custom RF surface coil into a preclinical 9.4 T MRI scanner (Fig. 1a). The multichannel optical imaging system consisted of a customized MRI-compatible fiberscope for delivering the excitation light and collecting the generated fluorescence (Fig. 1b). The fiberscope consists of an illumination bundle featured with 19 fibers with 600 µm core diameter and 0.4 numerical aperture (NA) for optimized coupling of two continuous wave (CW) lasers at 488 nm and 561 nm used for GCaMP and RCaMP excitation. Its additional component is a 1.4 mm diameter optic image guide consisting of 100,000 fibers collecting the emitted fluorescent responses through an objective (Fig. 1c, d). The fiberscope features an effective FOV of ~15 mm and spatial resolution of ~70 μm (Fig. 1d). The responses were split into two paths by a dichroic mirror to avoid signal crosstalk between the GCaMP and RCaMP fluorescence, which were subsequently detected by two dedicated cameras with a frame rate of 40 Hz. Note that by removing the emission filters, fluorescence imaging can readily be converted into intrinsic signal optical imaging (ISOI) mode. The HyFMRI data acquisition was synchronized with the stimulation paradigm using an external trigger device (see the “Methods” section for more details).

### Astrocyte and neuron labeling with viral transfection of calcium indicators

**Fig. 2:**
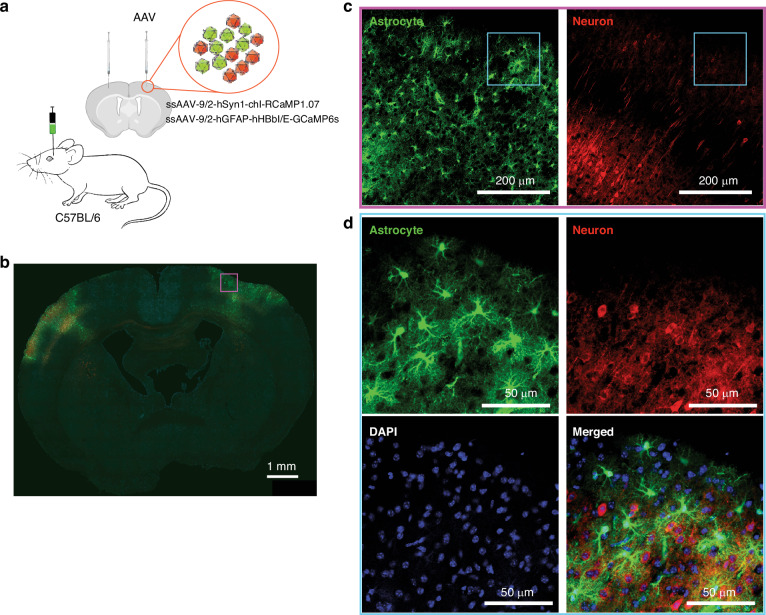
Intracranial AAV transfection of mixed GCaMP6s and RCaMP1.07 genes targeting astrocytes and neurons in C57BL/6 mice. **a** Illustration of the virus injection procedure. **b** Confocal fluorescence image of a representative ex vivo mouse brain slice showing widespread expression of the calcium indicators in both hemispheres. **c** Zoom-in view of the boxed region in (**b**) indicating the expression in astrocyte (green) and neuron (red). **d** Zoom-in view of the white boxed region in (**c**). Nuclei were counterstained by DAPI (blue)

Calcium indicator expression was achieved by adeno-associated viral (AAV) transfection of mixed GCaMP6s and RCaMP1.07 genes for astrocytes and neurons, respectively. The viruses were intracranially injected in C57BL/6 mice (Fig. 2a, see “Methods” for details on viral injection). Ex vivo imaging of mouse brain slices verified that calcium indicator expression was successfully induced in a large cortical area covering the somatosensory and motor cortices on both hemispheres (Fig. 2b). Magnified views into virally expressed regions revealed the densely distributed cells with high signal-to-noise ratio (SNR) and robust expression in neurons and astrocytes, further affirming a successful induction of cell-specific expression of calcium indicators (Fig. 2c, d).

### Image coregistration and brain parcellation

**Fig. 3:**
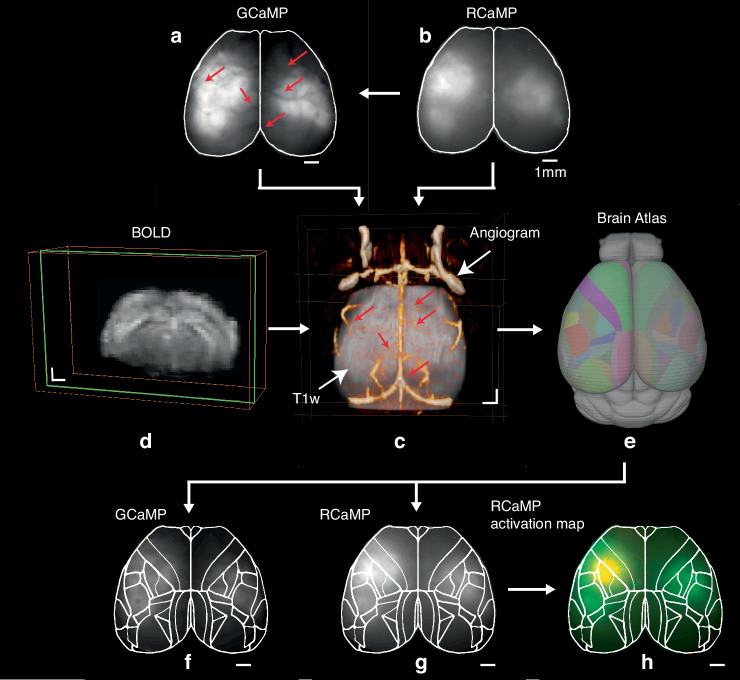
GCaMP and RCaMP image coregistration with fMRI BOLD scans and subsequent brain normalization to Allen mouse brain atlas and parcellation in transverse view. **a** GCaMP image. **b** RCaMP image. **c** Magnetic resonance angiography (MRA) image in yellow coregistered with T1-weighted (T1w) scan in gray of the same mouse brain. **d** A representative coronal slice of the fMRI BOLD image. **e** Allen mouse brain atlas with color-coded anatomical regions. **f**, **g** Corresponding GCaMP and RCaMP image with brain parcellation. **h** Calculated RCaMP activation map with brain parcellation. The hot spot indicates the activated region. Red arrows indicate vessels present in both FL and MRA. All scale bars: 1 mm

Coregistration between fluorescence images and fMRI BOLD data was performed indirectly due to a lack of common features. This was achieved by taking advantage of the common vascular information, brain outline and middle line in the fluorescence images (Fig. 3a, b) and magnetic resonance angiography (MRA) (Fig. 3c in orange). Despite the fact that label-free MRA presented limited information on the vascular network, it is sufficient to provide landmarks for coregistration. It is noted that the visibility of fiducial markers may be enhanced by recording additional optical intrinsic signal and contrast-enhanced MRA images of the subject, which however are not used in this work. To facilitate coregistration between fMRI BOLD scans (Fig. 3d) and MRA (Fig. 3c in orange), two T1-weighted (T1w) scans from the axial and coronal views were acquired as intermediates. Since the MRA and the T1w scan in axial view were acquired with the same geometry, they are inherently registered. Subsequently, the BOLD scans (Fig. 3d), which were already aligned to the T1w scan in coronal view, could be likewise precisely aligned to the T1w scan in axial view (i.e., the MRA, Fig. 3c) with SPM12 software based on mutual information. Owing to the established procedure for brain normalization with the Allen mouse brain atlas, GCaMP, RCaMP, and fMRI BOLD data can be readily aligned to a standard space (Fig. 3e, Supplementary Fig. S1). Furthermore, to enhance the correspondence and comparability with fluorescence images, a transverse plane fluorescence template was generated by projecting the cortical areas from layer 1 to 4 of the Allen mouse brain atlas for raw fluorescence images (Fig. 3f, g) and calculated activation map (Fig. 3h) brain parcellation.

### HyFMRI simultaneously captures neuronal, astrocytic and hemodynamic responses

**Fig. 4:**
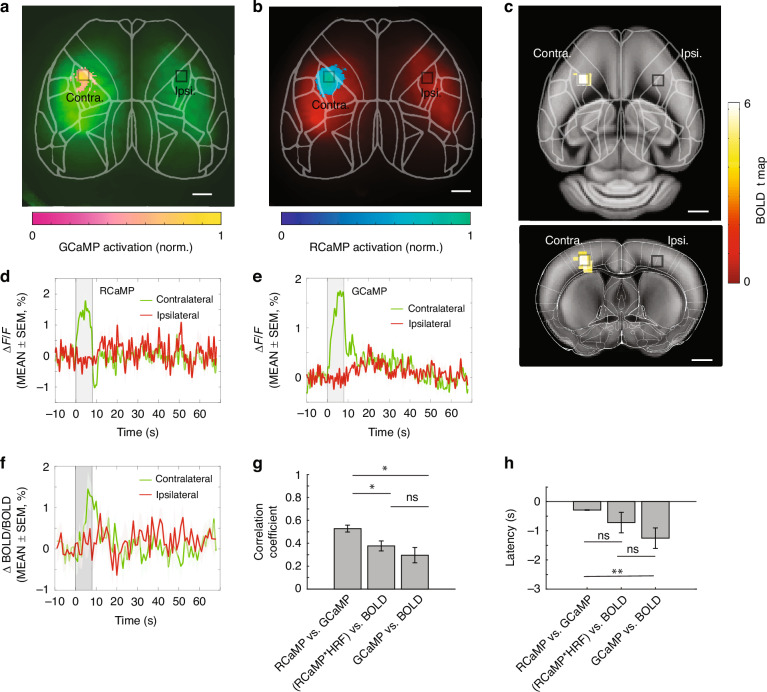
Brain responses to an electrical forepaw stimulation paradigm and corresponding signal time courses from a representative mouse. **a**–**c** Activation pattern from astrocytes (**a**), neurons (**b**) and hemodynamics (**c**). The activated area perfectly lies in the S1FL cortical region on the contralateral side, while no obvious activation was observed from the ipsilateral counterpart. For better visualization, BOLD activation map is overlaid on the Allen mouse brain atlas. **d**–**f** Signal time courses from RCaMP, GCaMP, and BOLD from the ROI in the activated region shown in panel (**a**)–(**c**), respectively. SEM standard error of mean. **g** Pearson’s correlation coefficient between RCaMP, GCaMP and BOLD time courses in all stimulation cycles, showing that GCaMP/RCaMP (0.5281 ± 0.0303) has a significantly higher correlation than RCaMP/BOLD (0.3769 ± 0.0435, *p* = 0.0115) and GCaMP/BOLD (0.2959 ± 0.0670, *p* = 0.0061). Comparison between RCaMP/BOLD and GCaMP/BOLD does not manifest significance (*p* = 0.3252). Two-tailed t test with a significance level of *p* < 0.05. (h) Response latency between RCaMP, GCaMP, and BOLD. GCaMP vs. RCaMP latency is 0.2889 ± 0.0094 s, significantly shorter than that of GCaMP vs. BOLD (1.2528 ± 0.3514 s, *p* = 0.0035) and shorter than that of (RCaMP*HRF) vs. BOLD (0.7194 ± 0.3522 s, *p* = 0.7123). The latency between GCaMP vs. BOLD and (RCaMP*HRF) vs. BOLD is not significantly different (*p* = 0.3077). Note that the data in all the three groups did not pass the normality test thus nonparametric Mann–Whitney test was performed. All scale bars: 1 mm. ns: not significant; *p < 0.05; **p < 0.005

To demonstrate the multiplexed imaging capabilities of the proposed platform, electrical forepaw stimulation was applied to a group of mice (*N* = 8) AAV transfected with GCaMP6s and RCaMP1.07 genes. Stimulus-evoked fluorescence responses from GCaMP and RCaMP as well as BOLD signals were recorded simultaneously (see Methods for details). HyFMRI successfully captured signals from the contralateral primary somatosensory cortex of forelimb (S1FL) corresponding to activation of neurons (1.8908 ± 0.1290%) and astrocytes (1.9578 ± 0.0780%), as well as hemodynamic responses (1.3436 ± 0.1609%) across the entire cortex (Fig. 4a–c). No obvious activation was observed in the ipsilateral hemisphere (Supplementary Fig. S2). Neuronal activations (Fig. 4d) demonstrated faster kinetics (time to peak, TTP: 3.8357 ± 0.5069 s, MEAN ± SEM) post-stimulation compared to astrocytic activity (6.6714 ± 0.3520 s, Fig. 4e), which were followed by the slower fMRI BOLD response (8.1111 ± 0.5879 s, Fig. 4f). To investigate temporal signal correlations between RCaMP, GCaMP and BOLD, Pearson’s correlation coefficients were calculated between RCaMP and GCaMP, predicted hemodynamic response from RCaMP convolved with hemodynamic response function (HRF) and BOLD, as well as GCaMP and BOLD during each stimulation cycle. It shows that RCaMP/GCaMP presents significantly higher correlation (0.5281 ± 0.0303) than RCaMP/BOLD group (0.3769 ± 0.0435, *p* = 0.0115) and GCaMP/BOLD group (0.2959 ± 0.0670, *p* = 0.0061), while the comparison between RCaMP/BOLD and GCaMP/BOLD groups does not show significant differences (*p* = 0.3252) using two-tailed t test (Fig. 4g), indicating a complex non-linear transformation between calcium and BOLD signals. Taking advantage of the concurrent recording capability, the stimulation evoked response latencies between RCaMP, GCaMP and BOLD were investigated by checking their cross-correlation. Interestingly, as compared to RCaMP, the GCaMP responses presented a short latency (0.28889 ± 0.0094 s) across the 9 stimulation cycles, while the latency between BOLD and GCaMP was more scattered (1.2528 ± 0.3514 s) (Fig. 4h). To assess the correlation between neuronal activity and the BOLD response, we further examined the latency between BOLD and the predicted hemoglobin response, which can be calculated by convolving the RCaMP response with a modified HRF (parameters: [2.3 16 0.34 1 6 0 8])^22^, assuming that hemodynamic responses can be modeled as a linear and time-invariant system. A latency of 0.7194 ± 0.3522 s was found (Fig. 4h), indicating a discrepancy of early response in the modified HRF.

HyFMRI reveals cell type-specific activation kinetics

**Fig. 5:**
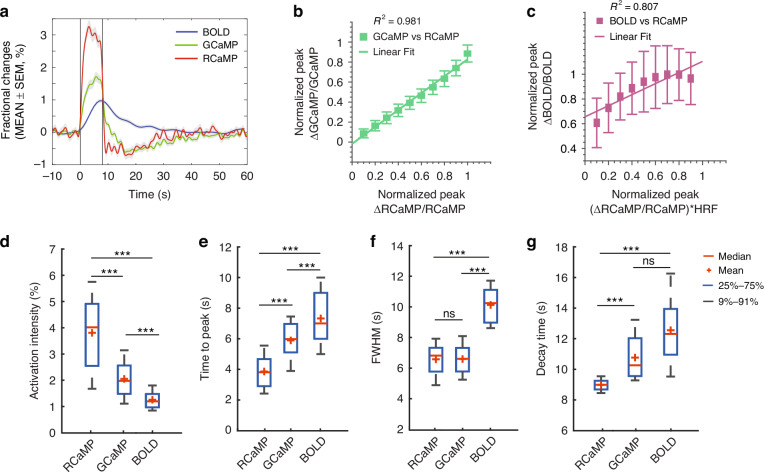
Activation kinetics evoked by electrical forepaw stimulation in the mouse brain. **a** Group-level (*N* = 8) BOLD, GCaMP, and RCaMP activation curves. **b** Correlation between GCaMP and RCaMP time courses. The linear fit of normalized activation peaks is shown. **c** Correlation between BOLD and predicted hemodynamic response by convolving RCaMP response with the modified HRF. The linear fit of normalized activation peaks is shown. **d**–**g** Statistics on the activation intensity, time-to-peak (TTP), response duration (FWHM), and decay time, which were extracted from the GCaMP, RCaMP, and BOLD time courses. For both activation intensity and TTP, differences between every two groups are significant (*p* < 0.0001). RCaMP and GCaMP exhibit significantly shorter responses as compared to BOLD (*p* < 0.0001) while no significant difference exists between response duration for RCaMP and GCaMP (*p* = 0.8121). RCaMP manifests significantly shorter decay time than GCaMP and BOLD (*p* < 0.0001) while no significant difference exists between GCaMP and BOLD (*p* = 0.0668). Two-tailed Mann–Whitney tests were performed between different groups. ns not significant; ***p < 0.0001

To investigate the activation kinetics to electrical forepaw stimulation, group level data analysis was performed (*N* = 8 mice). For this, we extracted average activation time courses from the S1FL region of each stimulation trial for GCaMP, RCaMP, and BOLD signals (Fig. 5a). Stimulation-evoked responses of RCaMP signal from neurons displayed the fastest kinetics and highest activation amplitude followed by GCaMP signal from astrocytes and the BOLD signal. By following the normalized activation time courses from each concurrently recorded stimulation cycles, GCaMP and RCaMP responses presented very high linear correlation (R^2^ = 0.981, Fig. 5b), thus providing direct evidence of astrocytic mediation in neurovascular coupling. We further assessed the correlation between BOLD responses with the predicted hemodynamic response by convolving the neuronal signal with the modified HRF for rodents, which revealed a reduced correlation due to a latency in the predicted responses (Fig. 5c). RCaMP signals presented significantly higher activation intensity (3.806 ± 0.157%, MEAN ± SEM) than GCaMP (2.051 ± 0.097%, *p* < 0.0001) and BOLD (1.257 ± 0.041%, *p* < 0.0001) (Fig. 5d). RCaMP signals also presented significantly faster response kinetics in terms of TTP (3.863 ± 0.122 s) than GCaMP (5.889 ± 0.161 s, *p* < 0.0001) and BOLD signals (7.329 ± 0.210 s, *p* < 0.0001) (Fig. 5e). On the other hand, BOLD signals presented significantly longer responses (10.120 ± 0.288 s at the full width at half maximum, FWHM) versus RCaMP (6.572 ± 0.123 s, *p* < 0.0001) and GCaMP (6.591 ± 0.134 s, *p* < 0.0001), while the comparison between RCaMP and GCaMP has not rendered significance (*p* = 0.8121). In terms of decay time defined as the duration from the peak to its baseline level, both BOLD (12.530 ± 0.998 s) and GCaMP (10.740 ± 0.222 s) presented significantly higher values as compared to RCaMP (8.953 ± 0.045 s, *p* < 0.0001 in both cases).

## Discussion

Brain exhibits a remarkable degree of spontaneous activity, making it challenging to apply spatial and/or temporal averaging strategies to interrogate neural activity. Subtle variations in cellular activity, such as transient changes in membrane potential or calcium signaling, as well as fluctuations in hemodynamic responses, including localized shifts in blood oxygenation and flow, serve as compelling evidence of true instantaneous changes in neural dynamics^23^. These variations are often obscured in serial or ensemble-averaged experiments, which fail to capture the stochastic and individualized nature of these events. In this work, by simultaneously recording multiple signals from different cell types, HyFMRI is shown capable of uncovering the nuanced interplay between neuronal, astrocytic, and vascular processes.

The role of astrocytes in neurovascular coupling has been intensively debated with growing attention to the neuron-astrocyte-vascular pathway. Current perspectives suggest that astrocytic calcium signaling not only plays a pivotal role in the physiology but also in pathology of the central nervous system, including the progression of neurodegenerative diseases such as neuroinflammation, synaptic dysfunction, and neuronal death^24^. While theoretical models exist to predict the role of astrocytes^25,26^, experimental validations and systematic investigations on the links between astrocytes and other brain cells remain scarce. HyFMRI addresses this critical gap by providing a straightforward and powerful platform to study the dynamic interplay between neurons, astrocytes, and vascular components at high temporal (40 Hz for fluorescence and 1 Hz for BOLD signals) and spatial (~70 μm for optical and 250–700 μm for BOLD) resolution. It is noted that strong scattering of fluorescence emission in the brain tissue compromises the effective spatial resolution of the optical measurements. This, however, does not affect the detection and localization of responses originating from a larger volume and subsequent data analysis. Stimulation-evoked neuronal responses from a 0.6 ×0.6 ×0.6 mm^3^ region in the activated area in the S1FL region displayed the fastest kinetics (TTP, 3.863 ± 0.122 s) and highest activation amplitude (3.806 ± 0.157%) followed by astrocytic signals (TTP, 5.889 ± 0.161 s; activation intensity 2.051 ± 0.097%) and the hemodynamic responses (TTP, 7.329 ± 0.210 s; activation intensity 1.257 ± 0.041%) retrieved by simultaneously recorded BOLD signals. The activation traces from neurons and astrocytes exhibited very high linear correlation (R^2^ = 0.981), providing direct evidence of astrocytic mediation in neurovascular coupling.

The ability to capture cell-type-specific calcium signaling alongside whole-brain hemodynamics offers new venues for investigating neuro-glial-vascular interactions in health and disease. This capability could enable the identification of pathophysiological deviations from normal spontaneous activity, thereby advancing our understanding of neurological disorders and cognitive dysfunctions. Furthermore, by bridging the gap between cellular-level activity and global brain function, HyFMRI could help validate theoretical models and expand the current understanding of the neuron-astrocyte-vascular axis.

HyFMRI holds several significant advantages over conventional photometry-based approaches and stand-alone widefield fluorescence imaging. First, it can measure cortex-wide neural activity simultaneously with fMRI, making it particularly suitable for brain function studies at the level of large networks or circuits. Second, HyFMRI is minimally invasive and easy to implement. This avoids significant drawbacks of photometry-based approaches, including unreliable stereotactic fiber positioning in young mice, which subsequently leads to poor SNR. In addition, regrowth of the skull may further degrade the signal quality. Last but not least, despite being limited by light scattering in the brain tissue, HyFMRI achieves a reasonable balance between spatial resolution and whole-cortical coverage. Although fiber photometry provides superior temporal resolution on a millisecond scale, it is not possible to spatially resolve structures as all photons emitted from the target region are typically integrated into a single signal in the fiber^14,27^. Recent advances in photometry have enabled multi-regional coverage as well as cortical layer-specific calcium recordings in mice^28,29^. However, neither approach is currently compatible with MRI. Compared to existing work on widefield fluorescence imaging and whole brain fMRI^21^, by discerning cell type-specific fluorescence measurements HyFMRI enables studies on neuron-glia interactions, which are essential for brain function and play a key role in neurological diseases. It also achieves better image quality with significantly simpler experimental workflow. Moreover, it does not require skull thinning, acute craniotomy or a long-term cranial window and thus can operate in a noninvasive or minimally invasive manner. Given its compact design, the proposed HyFMRI platform holds potential for future integration with other modalities, such as optoacoustic imaging, optogenetics, or transcranial ultrasound stimulation.

While combining widefield fluorescence imaging with fMRI offers the potential for cell-type-specific recordings of neural activity, several inherent limitations of fluorescence imaging restrict its broader applicability. Widefield fluorescence imaging is inherently two-dimensional, lacking depth-resolved information. Consequently, it is confined to imaging superficial brain regions, typically limited to cortical layers, which restricts its utility in deeper brain areas. Compared to multiphoton microscopy, widefield fluorescence imaging has broader spatial coverage but suffers from low spatial resolution, leading to spatial averaging as it records population-level signals from neurons and astrocytes. To address the lack of depth information in widefield fluorescence imaging, light field imaging techniques can be employed to achieve three-dimensional volumetric imaging with single acquisitions^30^. Combination with optoacoustic tomography can be an alternative solution for attaining whole-brain neural activity mapping with high spatial and temporal resolution^8,9^. To improve the spatial resolution of fluorescence imaging, structured illumination microscopy or localization microscopy techniques can be adopted to maintain high temporal resolution alongside large FOV^10,15,18,31–33^. To extend the penetration depth of excitation and emission light in biological tissue, the development of near-infrared-shifted calcium indicators, such as NIR-GECO, represents a significant advancement^34^. By further improving their labeling efficiency, these near-infrared genetically encoded calcium indicators are expected to facilitate the imaging of neural activity in deeper brain regions due to reduced scattering and absorption in this spectral window^35^.

In future work, the integration of optogenetics with HyFMRI holds great promise for advancing our ability to both modulate and simultaneously monitor neural activity with high precision. With light-sensitive ion channels especially in the cortical regions, optogenetic stimulation can be employed to activate or inhibit specific neural populations, while HyFMRI captures dynamic changes in cellular and whole-brain activity simultaneously, allowing the exploration of the causal relationships between neural activity and behavior, thus providing insights into how specific neuronal circuits contribute to cognition, sensory processing, or motor control. In addition, the synergy between local cellular activity and global network activity in the brain may allow for a more comprehensive understanding of how specific optogenetic manipulations impact large-scale brain function and connectivity.

In conclusion, we proposed the HyFMRI platform by integrating a fiberscope-based fluorescence imaging module with fMRI, enabling concurrent recordings of neuronal and astrocytic signals alongside hemodynamic responses. By monitoring the neural activity across spatial and temporal scales, this hybrid approach represents a powerful tool for advancing our understanding of brain function. It holds potential for investigating the mechanistic underpinnings of neurological and psychiatric disorders, such as depression, epilepsy, or Parkinson’s disease.

## Materials and methods

### Hybrid fluorescence and MRI (HyFMRI) platform

The HyFMRI platform is based on a customized multichannel fiber-optic imaging system and a customized RF surface coil integrated into a 3D-printed animal holder and inserted into a high-field MRI scanner (BioSpec 94/20, Bruker BioSpin, Germany) (Fig. 1a). The multichannel optical imaging system (Fig. 1b) consists of a fiberscope (Zibra Corporation, Westport, USA) used for delivering the excitation light and collecting fluorescence responses, two continuous wave (CW) lasers (488 nm and 561 nm, Sapphire LPX, Coherent, USA) for GCaMP and RCaMP excitation, and two cameras for fluorescence signal recording. The fiberscope consists of a 1.4 mm diameter optic image guide consisting of 100,000 fibers collecting the fluorescent responses through an objective (Lens1), and an illumination bundle composed of 19 fibers with 600 µm core diameter and 0.4 numerical aperture (NA) each for optical excitation. The collected fluorescence responses are separated into two channels by a dichroic mirror (DM2, Di03-R532-t1-25 × 36, Semrock). The GCaMP signal is then collected by an EMCCD camera (Camera1, Andor Luca, UK) after passing through the emission filter (Filter 1, FF01-525/39-25, Semrock) while RCaMP signal is collected by a CMOS camera (Camera2, Basler Ace2, Germany) after passing through the second emission filter (Filter 2, FELH0600, Thorlabs). Note that by removing the emission filters, one can easily convert fluorescence imaging to intrinsic signal optical imaging setup. The HyFMRI data acquisition is synchronized with the stimulation paradigm using an external trigger device (Pulse Pal V2, Sanworks, USA).

### Animal models

C57BL/6 mice (Charles River Laboratories, female, 7-8-week-old, *N* = 8) were used in this study. The animals were housed in individually ventilated, temperature-controlled cages under a 12-hour reversed dark/light cycle. Pelleted food (3437PXL15, CARGILL) and water were provided ad-libitum. Mouse housing, handling and experimentation were performed in accordance with the Swiss Federal Act on Animal Protection and were approved by the Cantonal Veterinary Office Zurich.

### Induction of calcium indicator expression

Adeno-associated viral (AAV) vectors containing GCaMP6s (AAV-9/2-hGFAP-hHBbI/E-GCaMP6s) and RCaMP1.07 (AAV-9/2-hSYN1-ch1-RCaMP1.07) genes were injected intracranially 3 weeks before the in vivo measurement to induce calcium indicator expression in astrocytes and neurons. Before injection, the stock solution of these two vectors was first mixed at 1:1 (v/v) with a physical titer of 1.0 × 1013 vg/ml for GCaMP and 4.3 × 1012 vg/ml for RCaMP. The injection procedure was performed under isoflurane anesthesia (5% induction, 1–2% maintenance). Two burr holes were drilled into the skull on two hemispheres (Bregma 0 mm, lateral 1.8 mm, depth 0.3 mm by an automatic drill (Ideal Micro Drill Surgical Drill, Harvard Apparatus, USA). The mixed virus was injected using a 10 μL syringe (NanoFil 10 μL Syringe, World Precision Instrument) and 33-gauge beveled needle (NF33BV, World Precision Instrument) using a hydraulic pump to inject the viruses (500 nL/spot at 50 nL/min) at a depth of ~300 μm into the somatosensory cortex, forepaw area on both hemispheres. During injection, the physiological status of the animal was constantly monitored, and the body temperature was kept around 37 °C. After injection, the needle was left in place for ~5 min before being slowly withdrawn with the scalp of the mouse sewed back afterwards. The animal was treated with buprenorphine (0.1 mg/kg) for 24 h following the surgery. The body weight of the animal was measured for three consecutive days post-injection and afterwards at least three times a week until the terminal experiment.

### In vivo imaging

All mice were anesthetized during the in vivo imaging experiments with intraperitoneal (i.p.) injection of a ketamine/xylazine mixture (100/10 mg/kg body weight for induction, 25/1.25 mg/kg body weight for maintenance). Maintenance injections were administered i.p. every 45 min. The scalp of the mouse was removed 20 min after injection of lidocaine (1%, 20 μl), while the skull was kept intact. Each mouse was positioned in the 3D-printed platform and ultrasound gel mixed with heavy water was applied on the mouse skull to ensure the moisture which is critical for optical imaging. The mouse head was immobilized using a custom 3D-printed stereotactic frame. After positioning the mouse, the platform containing both the fiberscope and the RF coil was inserted into the bore of the MRI scanner. During the experiment, an oxygen/air mixture (0.2/0.8 L.min^−1^) was provided through a breathing mask. Body temperature and respiration were continuously monitored during data acquisition with an MRI-compatible rectal thermometer and a pneumatic pillow (SA Instruments, USA). The heart rate and SpO_2_ were monitored in real-time with an MRI-compatible mouse paw pulse oximeter working with a PhysioSuite (Kent Scientific Corporation, USA). The body temperature was kept around 37 °C with a temperature-controlled water heating unit. After the experiment, the animals were euthanized while still under deep anesthesia.

### Sensory stimulation

For sensory stimulation, unipolar rectangular electric pulses of 0.5 ms duration and 0.5 mA intensity were applied to the left forepaw at 4 Hz stimulus frequency, 8 s onset time, and 82 s burst intervals, i.e., 90 s stimulus repetition cycle. The electric signals were generated using a stimulus isolator device (Model A365R, World Precision Instruments, USA) fed by an external trigger (Pulse Pal V2, Sanworks, USA). Each stimulation sequence included 9 stimulation cycles and lasted 900 s during which the first 82 s was reserved for baseline recording. The stimuli were synchronized with the concurrent camera acquisitions and MRI data acquisition.

### MRI data acquisition

MRI acquisitions were conducted on a 9.4 T small animal MRI scanner (Model: Biospec 94/20, Bruker BioSpin, Ettlingen, Germany) using a custom surface RF coil. ParaVision 6.0.1 was used as the user interface. Magnetic resonance angiogram (MRA) images were acquired to facilitate image coregistration with fluorescence data using a fast low angle shot (FLASH) sequence: FOV = 20 × 20 mm^2^, matrix dimension (MD) = 256 × 256, 20 slices from the brain surface to deeper regions, slice thickness = 0.3 mm, repetition time (TR) = 13 ms, echo time (TE) = 1.8904 ms, number of averages (NA) = 4. T1-weighted scan in the transverse plane with the same geometry as the MRA was performed to facilitate subsequent coregistration to the brain Atlas using a FLASH sequence: FOV = 20 × 20 mm^2^, MD = 128 × 128 × 20, 20 slices from the brain surface to deeper regions, slice thickness = 0.3 mm, TR = 500 ms, TE = 2.6203 ms, NA = 6. T1-weighted scan in the coronal plane was acquired as anatomical reference for the fMRI data using a FLASH sequence: FOV = 20 × 10 mm^2^, MD = 160 × 80, 11 slices from anterior to posterior, slice thickness = 0.7 mm, TR = 500 ms, TE = 2.1366 ms, NA = 8. Prior to fMRI data acquisition, the local field homogeneity was optimized using the acquired B0 field maps. BOLD data were acquired using a gradient-echo echo-planar imaging (GE-EPI) sequence: FOV = 20 × 10 mm^2^, MD = 80 × 40, yielding an in-plane voxel dimension of 250 × 250 µm^2^, 11 slices from anterior to posterior, slice thickness = 0.7 mm, flip angle (FA) = 60°, TR = 995 ms, TE = 12 ms, NA = 1, yielding an effective temporal resolution of 1 s for the volumetric acquisitions.

### Immunohistochemistry

Mice were anesthetized with a combination of ketamine (100 mg/kg)/xylazine (10 mg/kg)/acepromazine maleate (2–3 mg/kg) with a total injection volume of 100 μl and transcardially perfused with 2% paraformaldehyde (PFA). Brains were post-fixed in 4% PFA for 3 h and cryoprotected with 30% sucrose in phosphate-buffered saline (PBS) for 24 h. The brain was fixed in optimal cutting temperature (OCT) embedding compound and was cut with a freezing microtome and incubated with 4′,6-diamidino-2-phenylindole (DAPI) staining solution for 5 min. Images were acquired with a Zeiss Confocal LSM 800 microscope.

### Brain parcellation

Region definitions of the BOLD template for brain activation imaging were based on the Allen Common Coordinate Framework (CCFv3)^36^. The BOLD template was projected from 3D to 2D to generate the fluorescence template, focusing on cortical layers 1 to 4. This approach was chosen because the superficial cortical layers play a key role in the generation and propagation of neural signals detectable by fluorescence imaging^37^. For brain parcellation in the coronal view, the contours of different brain regions were extracted directly from Mouse Brain Atlas (https://labs.gaidi.ca/mouse-brain-atlas/) and overlaid onto the corresponding brain slice in the Allen mouse brain atlas.

### Data analysis

Functional data analysis was performed using Matlab (version R2019b, Mathworks, Natick, MA, USA) and the open-source SPM software (version 12, Functional Imaging Laboratory, Welcome Trust Centre for Human Neuroimaging, University College London). In detail, the first 5 s of the BOLD data were discarded prior to pre-processing. BOLD data were first aligned to the mean image of the sequence and then normalized to the Allen mouse brain atlas (Allen Institute for Brain Science, http://mouse.brain-map.org/). Motion estimation and correction were performed accordingly by using SPM12 Realign (estimate) function. Six motion parameters (i.e., three translations and three rotations) were obtained to perform a rigid-body transformation. Then BOLD data were resliced to a voxel size of 0.2 × 0.2 × 0.2 mm^3^ and smoothed by spatial convolution with a 0.6 mm FWHM Gaussian kernel. Fluorescence data were rearranged to form a 4D volume with the third dimension equal to 1 to make it compatible to the overall processing pipeline.

For the general linear model (GLM)-based 1-level analysis, default HRF parameters in SPM12 that are optimized for human studies were modified with a different set of parameters (i.e., [2.3 16 0.34 1 6 0 8]) tailored for a characteristic small animal BOLD response^22^. The six motion parameters obtained in the SPM12 motion correction step were also regressed to further reduce motion artifacts. A high-pass filter with a cut-on frequency of 1/200 Hz was used to remove slow signal drifts. Statistical significance of the event-evoked responses in the observations was evaluated with a contrast vector c = [1, 0]^T^. An experimentally measured neuronal response function was used to build the regressor for RCaMP data analysis^11^. Due to the unknown response function of astrocytes, a boxcar regressor was used for GCaMP data analysis. The t-map and *p*-values were calculated as statistical inferences. Note that, for rendering the activation map from BOLD, family-wise error rate (FWE) of *p* < 0.05 was applied to the t-map. For the fluorescence recordings, the parametric map (beta map) was used directly to infer the activation, as described in previous work^11^.

For the group-level analysis, activation curves were first averaged from single stimulation trials of eight mice. The activation curve from each stimulation trial was plotted from an ROI of 0.6 × 0.6 × 0.6 mm^3^ in the activated area in the S1FL region inferred by 1-level analysis in SPM12. Subsequently, GCaMP, RCaMP, and BOLD correlation analysis and statistics on activation parameters were performed, including activation intensity, response duration (FWHM), TTP, and decay time (T_1/2_, time duration from signal peak to baseline). To calculate the correlation between GCaMP, RCaMP, and BOLD signals from the representative mouse shown in Fig. 4h, Pearson’s linear correlation coefficient was first calculated for each stimulation cycle and then converted to z-score value using Fisher’s z-transformation. Similarly, in the group-level correlation analysis, signal time courses were correlated by checking the Pearson’s linear correlation coefficient, after being normalized to their corresponding peaks, to reveal additional information on the activation kinetics.

For visualization, BOLD activation map of each animal was overlaid onto the Allen mouse brain atlas. Activation maps from GCaMP and RCaMP recordings were overlaid onto the corresponding averaged image of that sequence. The time courses were analyzed within a time window covering 10 s pre-stimulation, 8 s stimulation onset, and 60 s post-stimulation. The baseline signal of each stimulation cycle was calculated by averaging the signals in the 10 s pre-stimulation time window. The fractional signal changes in each stimulation cycle were calculated as (signal(t)–baseline)/baseline, where t is time. The activation time course was obtained by averaging all the stimulation cycles.

### Statistics

To investigate the relationship between signal time courses from GCaMP, RCaMP, and BOLD, Pearson’s correlation coefficients were calculated during each stimulation cycle. To characterize the activation parameters on activation intensity, FWHM, TTP and decay time, data were extracted from each single activation curve from all the stimulation cycles from different mice. To test the difference between each group, taking into account the relatively small sample number, non-parametric two-tailed Mann–Whitney test was performed with a significance level of *p* < 0.05.

## Supplementary information

Supplementary File

## Data Availability

All data are available in the main text or the supplementary materials. Raw experimental data supporting the findings of this study are available from the corresponding author upon request.
