# Supplementary material for: Non-invasive large-scale imaging of concurrent neuronal, astrocytic, and hemodynamic activity with hybrid multiplexed fluorescence and magnetic resonance imaging (HyFMRI)
Source: Light Sci Appl. 2025 Sep 25;14:341. doi: 10.1038/s41377-025-02003-9 (PMC12462443; doi:10.1038/s41377-025-02003-9)
Supplement: Supplementary file 1 — Supplementary File [file 41377_2025_2003_MOESM1_ESM.pdf]

Supplementary Information for

Non-invasive large-scale imaging of concurrent neuronal, astrocytic, and hemodynamic activity with hybrid multiplexed fluorescence and magnetic resonance imaging (HyFMRI)

Zhenyue Chen[1,#], Yi Chen[2,3,#], Irmak Gezginer[2,3,#], Qingxiang Ding[1], Hikari AI Yoshihara[2,3], Xosé Luís Deán-Ben[2,3], Ruiqing Ni[2,3,4,5], and Daniel Razansky[2,3,4,*]

[1] Institute of Precision Optical Engineering, School of Physics Science and Engineering, Tongji University, Shanghai, China

[2] Institute for Biomedical Engineering and Institute of Pharmacology and Toxicology, Faculty of Medicine, University of Zurich, Switzerland

[3]Institute for Biomedical Engineering, Department of Information Technology and Electrical Engineering, ETH Zurich, Switzerland

[4]Zurich Neuroscience Center (ZNZ), Switzerland

[5]Department of Nuclear Medicine, Inselspital, Switzerland

[#]These authors contributed equally
*Correspondence to:
Daniel Razansky, Institute for Biomedical Engineering, Wolfgang-Pauli-Str. 27, 8093 Zurich, Switzerland
Email: daniel.razansky@uzh.ch

Figure S1 | Time series BOLD signal realignment using SPM 12 and its coregistration with Allen mouse brain atlas.

Figure S2 | GCaMP and RCaMP signal time series from the activated S1 region (contralateral side) and the ipsilateral hemisphere.

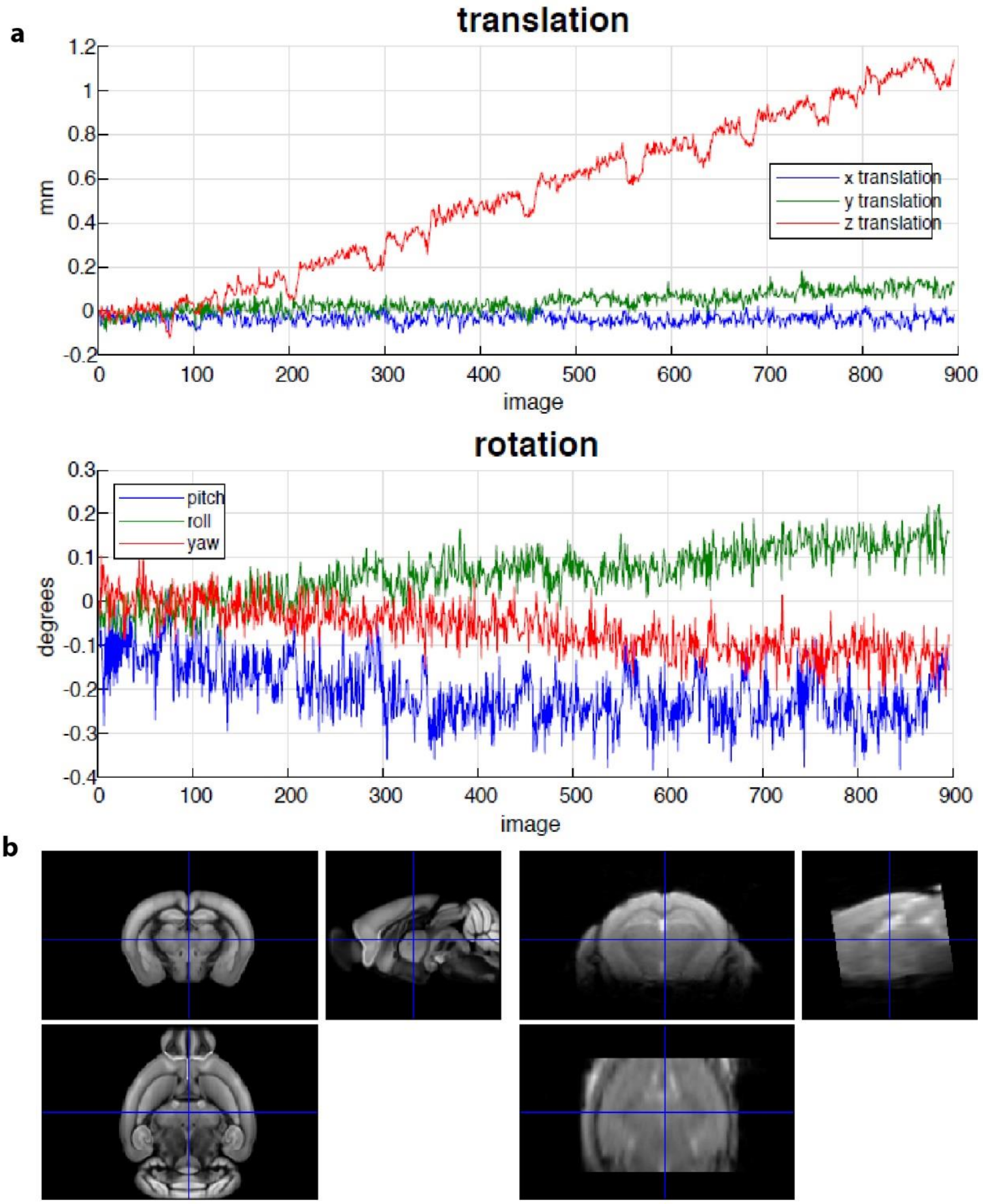

Figure S1 | Time series BOLD signal realignment using SPM 12 and its coregistration with Allen mouse brain atlas. (a) Translation and rotation parameters for BOLD realignment in the 900 s data sequence. (b) Coregistration between the Allen mouse brain atlas and averaged BOLD scan after realignment.

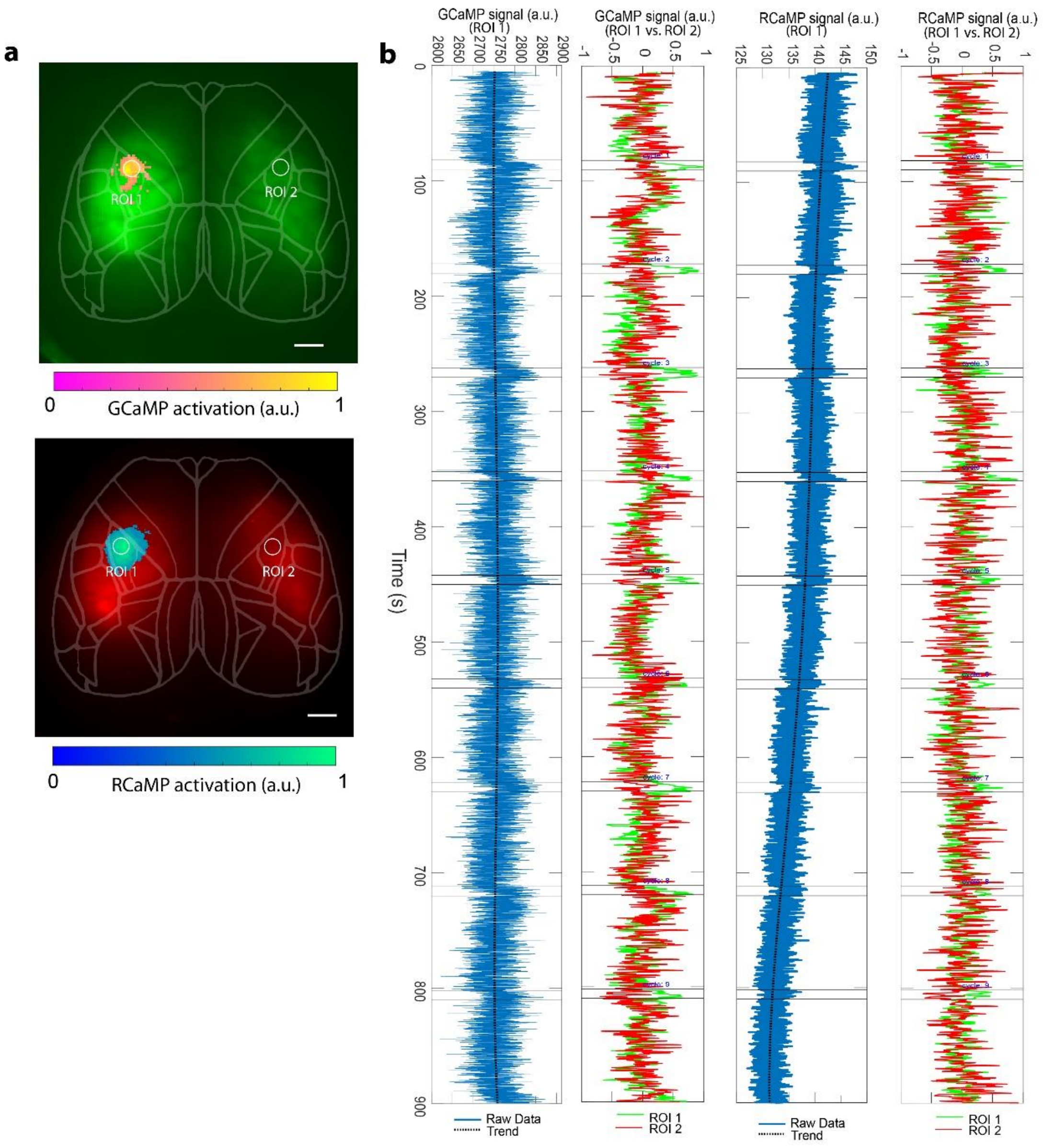

Figure S2 | GCaMP and RCaMP signal time series from the activated S1 region (contralateral side) and the ipsilateral hemisphere. (a) GCaMP and RCaMP activation map. (b) GCaMP and RCaMP signal time series from ROI1 and ROI2 shown in (a).
